# Boron Supplementation and Phytohormone Application: Effects on Development, Fruit Set, and Yield in Macadamia Cultivar ‘A4’ (*Macadamia integrifolia*, *M. tetraphylla*)

**DOI:** 10.3390/plants14162461

**Published:** 2025-08-08

**Authors:** Zhang-Jie Zhou, Zi-Xuan Zhao, Jing-Jing Zhou, Fan Yang, Jin-Zhi Zhang

**Affiliations:** 1 College of Horticulture and Forestry Science, Huazhong Agricultural University, Wuhan 430070, China; zhangjiezhou@webmail.hzau.edu.cn (Z.-J.Z.); zixuanzhao@webmail.hzau.edu.cn (Z.-X.Z.); hupodingxiangyu@mail.hzau.edu.cn (J.-J.Z.); 2 Institute of Tropical and Subtropical Cash Crops, Yunnan Academy of Agricultural Sciences, Baoshan 678000, China; 3 National Key Laboratory for Germplasm Innovation & Utilization of Horticultural Crops, Huazhong Agricultural University, Wuhan 430070, China

**Keywords:** macadamia, boron fertilizer, plant hormones, fruit set, regulatory mechanism

## Abstract

Macadamia (*Macadamia integrifolia*), *Macadamia tetraphylla* and hybrids, a crop of high economic and nutritional importance, faces challenges with low fruit set rates and severe fruit drop. To address this, we investigated the effects of exogenous plant growth regulators (PGRs) and boron fertilizer on the development, fruit set, and yield of the A4 macadamia variety. The study was conducted in 2024 at the Lujiangba research base (China, Yunnan Province). Five treatments were applied during key growth stages: boron (B), brassinosteroids (BR), N-(2-Chloro-4-pyridyl)-N’-phenylurea (CPPU), 6-benzylaminopurine (6-BA), and gibberellic acid (GA_3_). Growth stages include flower bud formation, peak flowering, and fruiting. Our findings revealed that B treatment significantly increased pollen viability (95.69% improvement) and raceme length (23.97% increase), while BR enhanced flower count per raceme (26.37% increase) and CPPU improved flower retention (10.53% increase). Additionally, GA_3_ and 6-BA promoted leaf expansion in new shoots, increasing leaf length by 39.83% and 31.39%, respectively. Notably, B application significantly improved total yield (43.11% increase) and fruit number (39.12% increase), whereas BR maximized nut shell diameter (5.7% increase) and individual nut weight (19.9% increase). Furthermore, CPPU and 6-BA markedly improved initial fruit set rates, while GA_3_, BR, and B effectively reduced early fruit drop. Physiological analyses indicated that elevated soluble sugars and proteins in flowers correlated with higher initial fruit set, whereas increased endogenous cytokinin and GA_3_ levels improved fruit retention and reduced drop rates. Based on these findings, we propose an integrated approach to optimize productivity: applying 0.02% B at the floral bud stage, 2 mg/L 6-BA at full bloom, and a combination of 0.02% B and 0.2 mL/L BR during early fruit set. This strategy not only enhances yield but also mitigates fruit drop, offering practical solutions for macadamia production.

## 1. Introduction

Macadamia (*Macadamia integrifolia Maiden* & Betche), *Macadamia tetraphylla* and hybrids, native to Australia, is a perennial evergreen tree belonging to the Proteaceae family and the Macadamia genus. It is also commonly known as the Hawaiian nut or Australian walnut [1]. Macadamia nuts are rich in nutrients, including soluble sugars, proteins, and unsaturated fatty acids [2,3]. They have been shown to regulate blood lipids, improve blood circulation, reduce the risk of cardiovascular diseases, and enhance memory, making them an excellent health food [4,5]. Macadamia represent a premium agricultural commodity with significant commercial value, enjoying sustained and growing demand in international markets owing to their distinctive nutritional composition and sensory characteristics [6]. The commercial cultivation of macadamia has a history of just over 150 years, with the first commercial plantations established in Hawaii. In the 1990s, macadamia cultivation was introduced to southern China for commercial planting [7]. China currently possesses the world’s largest macadamia cultivation area [8]. In Yunnan Province, the primary production region, soils are predominantly acidic (pH < 6.0) and characterized by low available boron levels, averaging merely 0.6 mg/kg—well below the optimal range of 1.0–2.0 mg/kg for macadamia cultivation [9]. Despite adequate soil concentrations of organic matter, alkali-hydrolyzable nitrogen, available copper, and zinc, growers consistently encounter production challenges including poor fruit set and severe premature fruit drop [10], which significantly constrain yield potential.

Large numbers of research has been conducted on the issue of macadamia fruit drop, including studies on the physiological patterns of fruit drop [11,12,13], the effects of pollinator varieties and pollination methods [14,15,16], measures to reduce fruit drop through the application of hormones and fertilizers [17,18,19], and the impact of water availability on fruit drop [20,21]. Recent research on the use of hormones to regulate fruit set and fruit drop has been conducted on various fruit trees, including apple [22], citrus [23], and jujube [24]. Additionally, research has been conducted on the effects of hormones, fertilization [25,26], and pollination [27] in macadamia. The hormones that have been studied include CPPU [11], GA_3_ [17], NAA [28], and others [29].

Cytokinins are a class of important plant hormones that play key roles in plant growth and development. Studies on endogenous cytokinins specifically have demonstrated their crucial functions in these processes: primarily promoting cell division and differentiation to drive growth [30]. Additionally, endogenous cytokinins regulate leaf growth and delay senescence [31,32], enhance photosynthesis [33], promote bud development and branching [34], improve the organogenesis ability of plant explants [35,36], modulate root morphogenesis [37], and mediate plant responses to stress, including drought, salinity, and pathogen defense [38]. CPPU, a synthetic exogenous cytokinin, enhances fruit set and size. Zeng et al. found that foliar application of CPPU (20 mg/L) 15 days after flowering significantly promoted early fruit set in macadamia [11,39]. CPPU treatment led to a dynamic pattern of the fruit drop rate, characterized by an initial increase, followed by a decrease, and then another rise. Moreover, CPPU treatment effectively reduced the rate of young fruit drop. Mechanistically, by enhancing carbohydrate utilization and maintaining a balance of endogenous hormones, CPPU was able to reduced early fruit drop [39].

Gibberellins (GAs), such as GA_1_, GA_3_, and GA_7_, are phytohormones biosynthesized by plants, ubiquitously distributed in plants. GAs regulate multiple aspects of plant growth and development through distinct mechanisms. For example, as key endogenous regulators, they enhance cell elongation and division to promote stem extension; activate hydrolytic enzymes like amylase to stimulate seed germination; modulate flowering time and improve fruit set; augment fruit expansion and quality while regulating ripening processes; and delay senescence through hormonal balance modulation [40]. Previous studies have validated these functions across species. Liu et al. reported that 20 mg/L GA_3_ treatment significantly enhanced pollen viability of macadamia in the Kau, Pahala, and Makai cultivars, demonstrating elevated germination rates and extended pollen tube growth compared to controls [17]. Conversely, Zeng et al. observed that exogenous GA application in macadamia induced paradoxical effects, promoting vegetative growth while suppressing floral development [41]. Complementing these findings, Liu et al. demonstrated practical agricultural benefits, showing that foliar application of 20 mg/L GA_3_ during full bloom significantly increased macadamia nut yield [42].

Auxins are pivotal regulators of plant growth and development [43]. A primary function is their induction of polarity and establishment of gradients. Their multifaceted roles are manifested through the following several mechanisms: As a phytohormone with pleiotropic effects, endogenous auxin orchestrates developmental processes through cell type-specific actions [44]. Additionally, auxin originating from shoot and root sources mediates phototropism and gravitropism. This occurs through its asymmetric redistribution in response to light and gravity, directing shoot growth towards light and root growth towards soil substrates [45]. Beyond these functions, endogenous auxins synthesized in the root pericycle and shoot axillary meristems stimulate branching and lateral root proliferation by activating lateral bud development, thereby enhancing root system complexity and nutrient absorption capacity [46,47]. During fruit development, endogenous auxins produced in developing embryo and endosperm regulate fruit development by accelerating growth, maturation, and abscission control [48]. When applied exogenously, these hormones demonstrate agricultural utility in reducing premature fruit drop and improving yield stability in crops [49,50]. Notably, macadamia cultivation benefits from auxin applications for flower induction, fruit retention, and quality enhancement. For example, a single post-flowering treatment with 1 ppm NAA (a synthetic auxin analog) increased young fruit count by 35% [28]. Further supporting this, foliar application of 20 mg/L of NAA during early fruit development significantly elevated macadamia yield, first-grade kernel ratio, and individual fruit weight [42].

In addition to hormonal regulation, emerging research highlights the critical role of boron fertilization in modulating macadamia yield. As an essential micronutrient, boron serves as a pivotal regulator of auxin transport in plants while simultaneously influencing multiple physiological processes [51]. Being non-reusable in plants, it necessitates seasonal soil application to maintain optimal function [52]. Boron participates in critical physiological processes, such as root and shoot apical meristem elongation, carbohydrate metabolism, sugar translocation, floral and fruit development, and pollen germination and pollen tube elongation—all vital mechanisms influencing fruit set efficiency and crop productivity [53]. In macadamia, the boron content typically ranges between 5.0 and 12.0 mg/kg [54]. Stephenson’s experimental trials demonstrate that foliar application of 0.02% boron solution at monthly intervals during nut development phases can induce statistically significant yield enhancements, underscoring the nutrient’s practical importance in commercial orchard management [55]. Although the research on the effects of hormone and boron fertilizer on macadamia has a certain basis, the research on the effects of hormone and boron fertilizer application in different key growth periods is relatively rare, and there is a lack of comparison of the effects of multiple hormones and boron fertilizer application under the same conditions. This study examines the impact of plant growth regulators and boron fertilizer sprays on macadamia trees by measuring morphological and physiological indicators during key stages: flower bud formation, peak flowering, and fruiting. Specifically, we aim to assess how exogenous hormones and boron affect development, determine optimal treatments to boost yield and fruit quality by improving fruit set and reducing drop rates, and examine treatment impacts on soluble sugars, proteins, and endogenous hormones in macadamia organs. The findings of this study will provide valuable insights for optimizing macadamia production through targeted hormonal and nutritional interventions.

## 2. Results

### 2.1. Effects of Boron and Exogenous Hormones on Shoot and Leaf Morphology

To investigate the effects of boron fertilizer and exogenous hormones on the morphology of new shoots and leaves, we measured leaf parameters (length, width, and aspect ratio) and new shoot traits (length, node count, and first internode length) under various treatments. The treatments for the experimental group included the following: 80.7 μM CPPU; 8.88 μM 6-BA; 57.8 μM GA_3_; 107 μM NAA; 0.416 μM BR; 500 μM B; and a mixed-solution containing all components at specified concentrations (80.7 μM CPPU + 8.88 μM 6-BA + 57.8 μM GA_3_ + 107 μM NAA + 0.416 μM BR + 500 μM B). Comparative analysis revealed distinct treatment effects: GA_3_, 6-BA, and boron fertilizer each significantly increased leaf length in new shoots compared to the control, with GA_3_ demonstrating the strongest elongation effect (+ 39.83% vs. control). Leaf width showed significant enhancement under 6-BA (+41.80%), GA_3_ (+33.86%), and BR (+33.33%) treatments, following the hierarchy 6-BA > GA_3_ > BR. The leaf aspect ratio exhibited marked variation, reaching its maximum under CPPU treatment (+5.74% vs. control) and its minimum with BR application (−12.53% vs. control). Regarding shoot development, boron fertilizer (−21.35%) and BR (−21.74%) significantly suppressed total shoot elongation relative to the control, while NAA resulted in longest shoot (+9.32% vs. control). Notably, all treatment groups showed non-significant variation from the control in both node count and first internode length (Table 1). Additionally, different treatments had no significant impact on leaf morphology (Table S2).

### 2.2. Effect of Boron Fertilizer and Exogenous Hormones on Raceme and Pollen Viability

**Figure 1:**
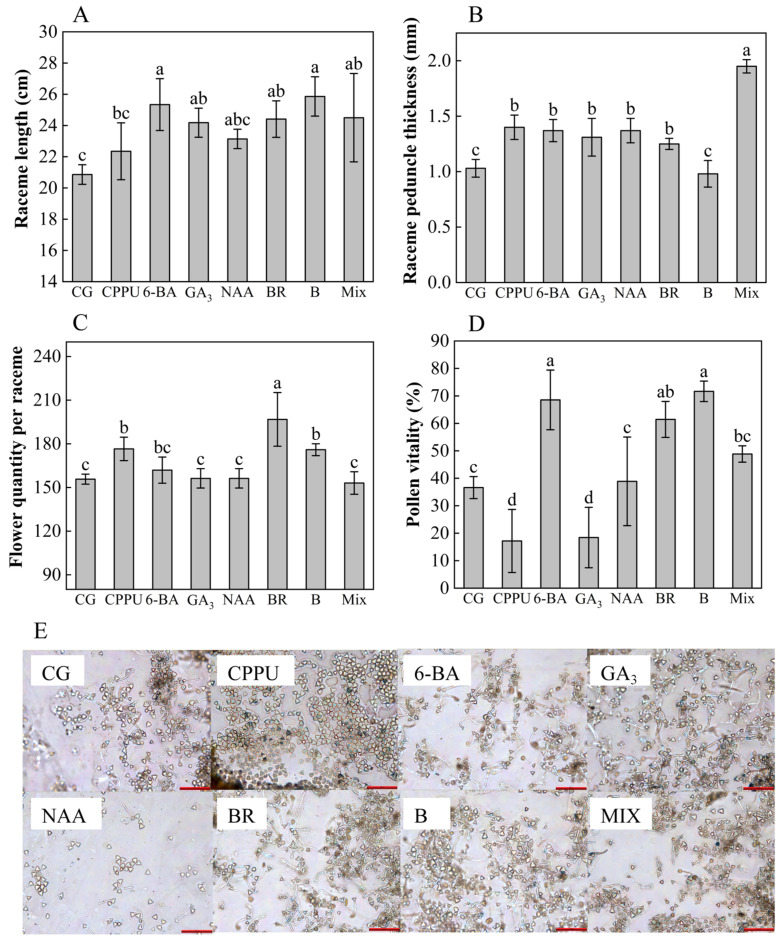
Raceme length, peduncle thickness, number of flowers per raceme, pollen viability, and pollen germination. (**A**) Raceme length (cm); (**B**) raceme peduncle thickness (mm); (**C**) flower quantity per raceme; (**D**) pollen viability (%). (**E**) Pollen images from trees under water control (CG), CPPU, GA_3_, NAA, boron fertilizer (**B**), mixed-solution (MIX), BR, and 6-BA treatments. Scale bar: 200 μm (red, lower right). Data represent means ± SD (n = 3). Different letters (a, b, c) indicate significant differences among groups (*p* < 0.05, Duncan’s test).

To evaluate the impacts of boron fertilizer and exogenous hormone treatments on raceme development, key reproductive traits—including pollen viability, raceme morphology (length and peduncle thickness), and the number of flowers per raceme—were measured. The findings revealed distinct treatment-specific responses across the measured parameters. Boron fertilizer emerged as the most effective intervention for raceme elongation, significantly surpassing the water control (+23.97% vs. control) and outperforming other treatments (6-BA, mixed-solution, BR, and GA_3_) in sequential order of efficacy (Figure 1A). In contrast, peduncle development followed a different pattern: the mixed-solution treatment induced the thickest peduncle in the raceme (+89.32% vs. control), exceeding the effects of CPPU, 6-BA, NAA, GA_3_, and BR (Figure 1B). The number of flowers showed particular sensitivity to BR application, which produced 26.37% more flowers per raceme than the control and surpassed both CPPU and boron fertilizer treatments (Figure 1C). Pollen viability exhibited dramatic variation across treatments, with boron fertilizer achieving a remarkable 95.69% increase in viability relative to the control. While 6-BA (+87.19%) and BR (+67.75%) maintained positive effects, GA_3_ (−49.67%) and CPPU (−53.08%) unexpectedly reduced pollen germination rates (Figure 1D). Microscopic evaluations corroborated these trends, demonstrating optimal pollen germination patterns in boron- and 6-BA-treated specimens (Figure 1E). These differential responses highlight the complex interplay between nutrient supplementation and hormonal regulation in macadamia reproductive development.

### 2.3. Physiological Responses of Flowers Under Different Treatment Conditions

**Figure 2:**
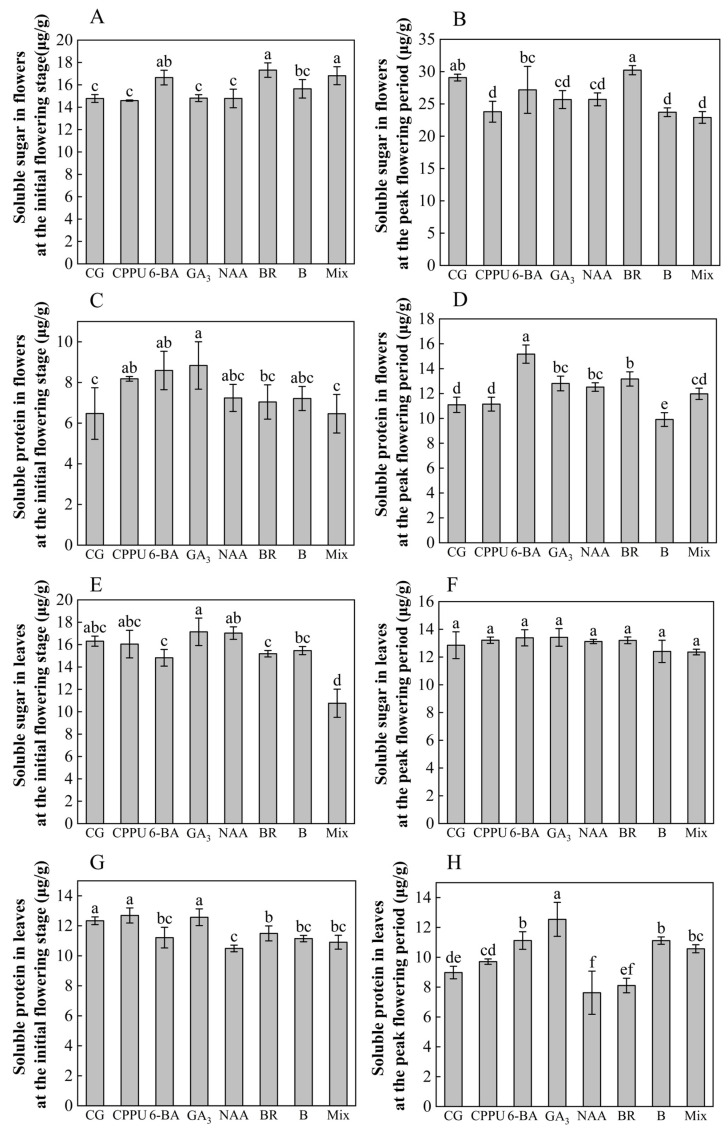
Physiological indicators of flowers and leaves. (**A**) Soluble sugar in flowers at flowering stage; (**B**) soluble sugar in flowers at peak flowering period; (**C**) soluble protein in flowers at flowering stage; (**D**) soluble protein in flowers at peak flowering period; (**E**) soluble sugar in leaves at flowering stage; (**F**) soluble sugar in leaves at peak flowering period; (**G**) soluble protein in leaves at flowering stage; (**H**) soluble protein in leaves at peak flowering period. Data represent means ± SD (n = 3). Different letters (a, b, c, d, e, f) indicate significant differences among groups (*p* < 0.05, Duncan’s test).

To investigate the physiological regulation mechanisms underlying raceme development during flowering, we analyzed soluble sugar and protein dynamics in flowers and leaves across initial and peak flowering stages. At the flowering stage, BR, mixed-solution, and 6-BA treatments significantly elevated soluble sugar content in flowers, with BR demonstrating the most pronounced enhancement (+17.19% vs. control) (Figure 2A). Conversely, GA_3_, CPPU, and 6-BA stimulated soluble protein accumulation, reaching peak levels under GA_3_ treatment (+36.48% vs. control) (Figure 2B). During peak flowering, BR-maintained flowers retained significantly higher soluble sugar content (+3.96% vs. control) compared to other treatments that fell below control values (Figure 2C). Meanwhile, 6-BA (+36.70%), BR (+18.76%), GA_3_ (+15.51%), and NAA (+12.89%) significantly boosted floral soluble protein levels, contrasting with boron fertilizer’s suppressive effect (−10.64%) (Figure 2D).

In leaf tissues during flowering, the mixed-solution reduced soluble sugar accumulation (−34.09% vs. control) (Figure 2E), while BR (−6.89%), 6-BA (−9.16%), boron (−9.72%), mixed-solution (−11.58%), and NAA (−14.99%) collectively decreased soluble protein content (Figure 2F). At peak flowering, leaf soluble sugar showed no significant inter-treatment variation (Figure 2G), whereas soluble protein dynamics diverged: NAA depressed protein levels (−15.44%) while GA_3_ (+39.64%), 6-BA (+23.83%), boron (+23.83%), and mixed-solution (+10.57%) enhanced them (Figure 2H). These stage-specific and tissue-dependent responses highlight the complex regulatory interplay between phytohormones and nutrients during reproductive development.

### 2.4. Effects of Boron and Exogenous Hormones on Fruit Abscission

**Figure 3:**
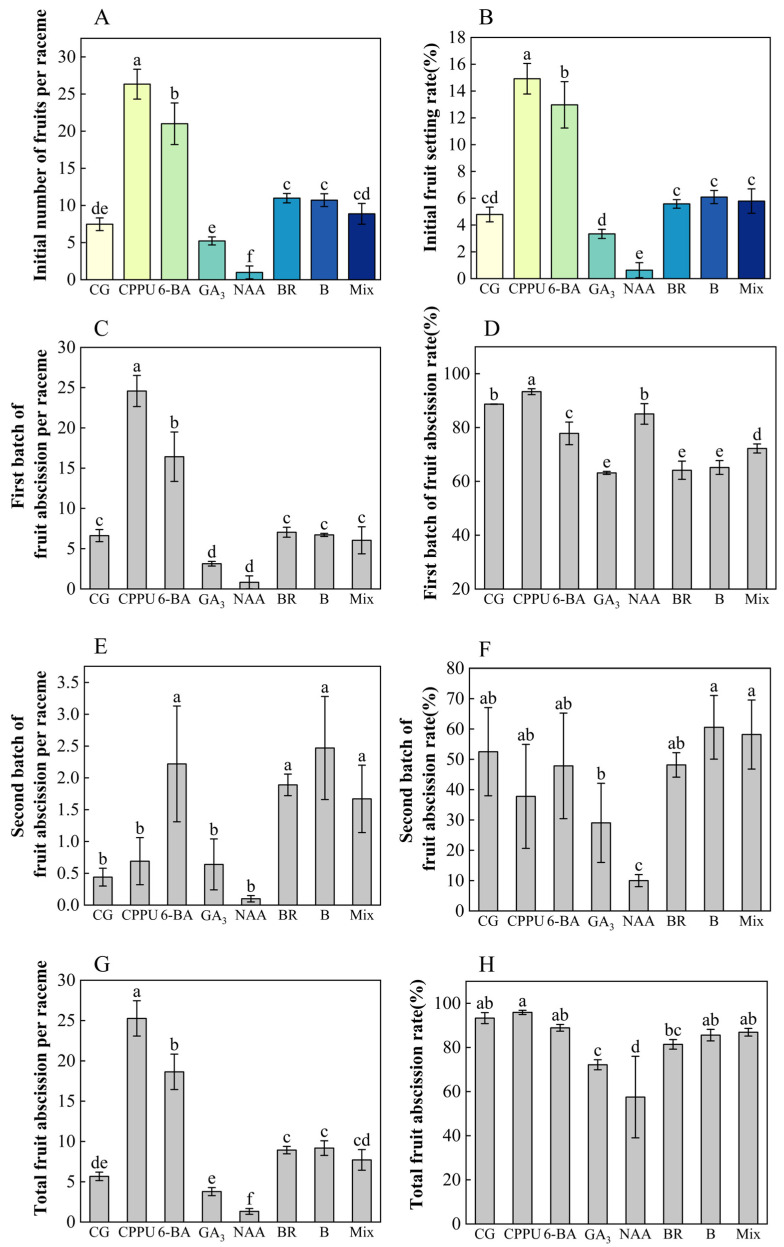
Fruit abscission and abscission rates under different treatments. (**A**) Initial fruit set (number per raceme); (**B**) initial fruit set rate (%); (**C**) first-batch fruit abscission per raceme (weeks 1–3); (**D**) first-batch fruit abscission rate (weeks 1–3); (**E**) second-batch fruit abscission per raceme (week 4 to week 20); (**F**) second-batch fruit abscission rate (week 4 to week 20); (**G**) total fruit abscission per raceme (fruit set to maturity); (**H**) total fruit abscission rate (fruit set to maturity). Data represent means ± SD (n = 3). Different letters (a, b, c, d, e, f) indicate significant differences among groups (*p* < 0.05, Duncan’s test).

To investigate the effects of boron fertilizer and exogenous hormones on the fruit setting, we recorded the initial number and rate of fruit setting per raceme, as well as the weekly number and rate of fruit setting per raceme during the first four weeks after fruit setting. Meanwhile, we measured the number and rate of fruit abscission per raceme weekly, along with the cumulative number and rate of fruit abscission per raceme over the study period. Among these treatments, CPPU, 6-BA, BR, and boron fertilizer all markedly improved the initial fruit set per raceme and fruit set rate compared to the water control. Notably, CPPU treatment yielded the most substantial enhancements, elevating initial fruit set per raceme by 252.47% and fruit set rate by 211.48%. The efficacy ranking remained consistent across all measured parameters, with treatments ordered as follows: CPPU > 6-BA > boron = mixed-solution = BR > water > GA_3_ > NAA (Figure 3A,B).

Boron and hormone treatments exhibited distinct temporal effects on fruit abscission patterns. Early-phase abscission (0–3 weeks post-treatment) was most pronounced under CPPU and 6-BA treatments. During the peak abscission period, CPPU further increased fruit abscission, while boron, BR, and GA_3_ significantly reduced the fruit abscission rate compared to the control (showing respective reductions of 28.8%, 27.7%, and 26.6% relative to the water control) (Figure 3C,D).

During the mid-stage of abscission (2–3 weeks), treatments with 6-BA, BR, boron, and CPPU maintained higher fruit setting rates, with 6-BA showing the most significant effect (Figure S1). Notably, although the 6-BA treatment showed significantly lower first-batch of fruit abscission rate than the control (18.77% reduction) (Figure 3D), both 6-BA and CPPU treatments induced higher fruit abscission quantities during the first two weeks after fruit setting compared to other treatments (Figure S1D,E). In the late stage of abscission, NAA had the lowest fruit abscission rate (80.95% reduction vs. control), while boron, 6-BA, and BR resulted in higher rates (Figure 3E,F). Furthermore, CPPU and 6-BA significantly increased the total fruit abscission rate (with CPPU exhibiting the highest abscission rate and NAA the lowest), whereas GA_3_ (−22.64%) and NAA (−38.36%) effectively mitigated the increase in total fruit abscission rate (Figure 3G,H).

### 2.5. Effects of Boron and Exogenous Hormones on Fruit Development

**Figure 4:**
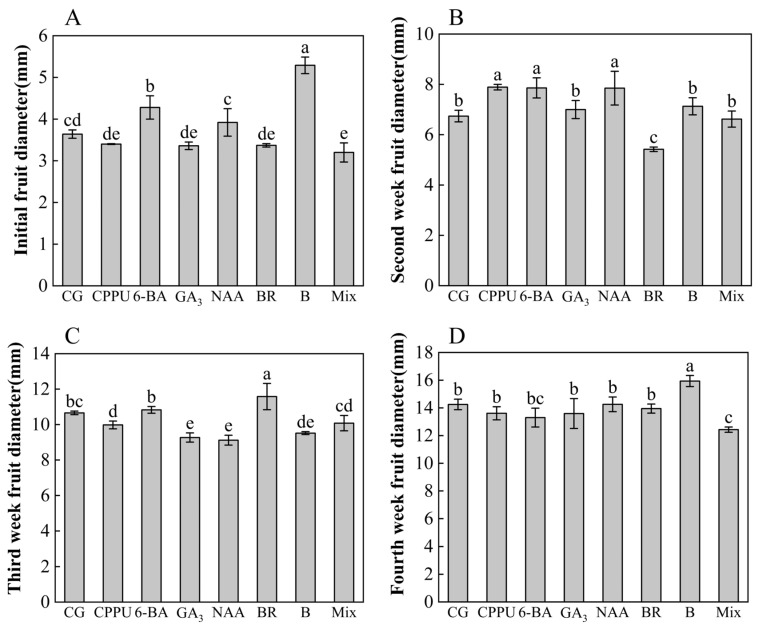
Fruit diameter during first four weeks after different treatments. (**A**) Diameter at initial fruit set (first week); (**B**) diameter at second week; (**C**) diameter at third week; (**D**) diameter at fourth week. Data represent means ± SD (n = 3). Different letters (a, b, c, d, e) indicate significant differences among groups (*p* < 0.05, Duncan’s test).

To investigate the effects of different treatments on fruit size kinetics, this study examined temporal changes in the transverse diameter of fruits during the first four weeks after fruit setting. As illustrated in Figure 4A–D, distinct growth patterns emerged across treatments and developmental stages. During the first week, boron- and 6-BA-treated fruits exhibited the largest dimensions, with increases of 45.33% and 17.58%, respectively, compared to the control, while those subjected to the mixed-solution consistently displayed the smallest size (Figure 4A). A growth hierarchy shift occurred in the second week, with CPPU-treated fruits demonstrating superior expansion (17.06% increase vs. control) compared to other treatments, whereas BR-treated fruits showed minimal growth (Figure 4B). The third week marked a reversal, as BR-treated fruits unexpectedly manifested the highest growth rate, though this stimulatory effect proved transient (Figure 4C). By the fourth week, boron re-emerged as the most effective treatment for maximizing fruit size (11.86% increase vs. control) (Figure 4D). Temporal analysis revealed treatment-specific growth trajectories: CPPU and NAA induced rapid early expansion during the initial week, while BR-mediated growth peaked in the second week before declining. Cumulative growth assessments over the three-week observation period (Figure S2) demonstrated boron’s sustained promotive effects, yielding the greatest overall expansion. Conversely, mixed-solution and 6-BA treatments consistently produced the smallest cumulative growth, suggesting either inhibitory interactions between components or suboptimal concentration ratios in these applications.

### 2.6. Hormonal Dynamics in Response to Different Treatments During Flowering Stages

To investigate the effects of boron fertilizer and exogenous hormones on endogenous hormones in flowers, the content of endogenous hormones was measured during the initial and peak flowering stages. At the initial flowering stage, GA_3_ and 6-BA treatments significantly increased ZEA (zeatin) content, with GA_3_ showing the highest levels (+53.85% vs. control), followed by 6-BA > BR > control > NAA = boron > CPPU > mixed-solution. By the peak flowering stage, 6-BA and BR treatments were the most effective in enhancing ZEA levels (with increases of 31.91% and 14.89% vs. control), with 6-BA exhibiting the highest concentration, followed by BR > GA_3_ > control = boron > CPPU = NAA > mixed-solution (Table 2).

In flowers, GA_1_ content was initially reduced across all treatments, whereas GA_3_ levels increased under the mixed-solution and GA_3_ treatments. At the peak flowering stage, GA_3_ treatment elevated both GA_1_ and GA_3_ content. For auxins, 6-BA and boron treatments initially enhanced IAA levels (+181.37% and +37.85% vs. control), with 6-BA being the most effective (6-BA > boron > control = CPPU = GA_3_ = BR = mixed-solution = NAA). Additionally, the mixed-solution and boron treatments increased ICA (indole-3-carboxylic acid) levels (by 144.44% and 87.30% vs. control). At the peak flowering stage, the mixed-solution, BR, 6-BA, and GA_3_ treatments elevated IAA content, ranked as follows: mixed-solution = BR = 6-BA > GA_3_ > control = CPPU = NAA = boron. The mixed-solution also increased ICA levels (by 68.52% vs. control). ABA content was higher in most treatments at the initial flowering stage, with NAA showing the greatest accumulation (+238.29% vs. control) (NAA > mixed-solution = CPPU > BR = 6-BA > boron > GA_3_ = control) (Table 2).

At the peak flowering stage, BR treatment led to the highest ABA levels (+32.93% vs. control), followed by NAA > mixed-solution = 6-BA = CPPU > control > boron = GA_3_. For jasmonic acid (JA), 6-BA treatment initially increased JA levels most prominently (+54.42% vs. control) (6-BA > boron > NAA = mixed-solution > control > CPPU > BR = GA_3_). However, at the peak flowering stage, CPPU and the mixed-solution dominated JA accumulation, with CPPU being the most effective (+56.04% vs. control) (CPPU > mixed-solution > BR > GA_3_ > control = 6-BA > boron > NAA). Finally, salicylic acid (SA) levels were initially elevated by the mixed-solution and CPPU treatments, with increases of 65.34% and 43.56% vs. control, ranked as follows: mixed-solution > CPPU > GA_3_ > BR = 6-BA = control > NAA > boron. These findings demonstrate dynamic and stage-specific hormonal responses to different phytohormone treatments during flowering (Table 2).

### 2.7. Effects of Boron and Exogenous Hormones on Fruit Morphology

**Figure 5:**
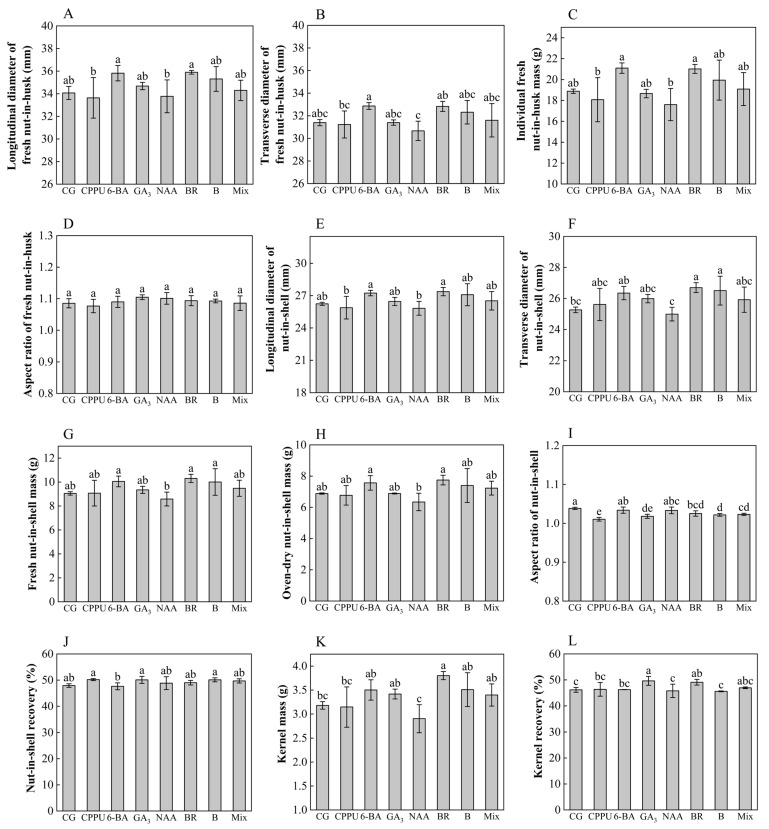
Effects of boron and exogenous hormones on fruit morphology. (**A**) Longitudinal diameter of fresh nut-in-husk (mm); (**B**) transverse diameter of fresh nut-in-husk (mm); (**C**) individual fresh nut-in-husk mass (g); (**D**) length-to-diameter ratio (aspect ratio) of fresh nut-in-husks. (**E**) Longitudinal diameter of nut-in-shell (mm); (**F**) transverse diameter of nut-in-shell (mm); (**G**) fresh weight of individual nut-in-shell (g); (**H**) oven-dry weight of individual nut-in-shell (g); (**I**) length-to-diameter ratio (aspect ratio) of nut-in-shell. (**J**) Nut-in-shell recovery (%); (**K**) oven-dry weight of individual kernel; (**L**) kernel recovery. Data represent means ± SD (n = 3). Different letters (a, b, c, d, e) indicate significant differences among groups (*p* < 0.05, Duncan’s test).

To investigate the effects of boron fertilizer and exogenous hormones on mature fruit morphology, comprehensive measurements were conducted on fresh nut-in-husk (green-skinned fruits) and nut-in-shell (shelled fruits), focusing on transverse/longitudinal diameters, aspect ratio, single fresh/dry nut-in-shell mass, nut-in-shell recovery, kernel mass, and kernel recovery. The experimental results demonstrated significant treatment-specific impacts on fruit morphology (Figure 5). While fresh nut-in-husk exhibited no statistically meaningful variations in dimensional parameters (transverse/longitudinal diameter) or weight metrics compared to the water controls (Figure 5A–D), distinct morphological responses emerged in nut-in-shell. Both BR and boron fertilizer treatments notably enhanced husked fruit transverse diameter, achieving maximum expansion of 5.7% relative to the controls, with BR showing superior efficacy (Figure 5F). This increase in diameter did not cause significant changes in the fresh or dry quality of individual nut-in-shell (Figure 5G,H). Likewise, both the longitudinal diameter of nut-in-shell and nut-in-shell recovery showed no statistically significant changes (Figure 5E,J). The most striking morphological modification occurred under CPPU application, which reduced the nut-in-shell aspect ratio by 9.2% (Figure 5I), producing characteristically compact fruits contrasting with the elongated control morphology. Kernel weight analysis demonstrated that brassinolide significantly increased single-kernel mass (by 19.5% vs. control) (Figure 5K), while the GA_3_ and brassinolide treatments markedly enhanced kernel recovery (by 7.43% and 6.32%, respectively, vs. control) (Figure 5L). These differential responses highlight the compound-specific nature of phytohormonal effects on fruit development parameters.

### 2.8. Effects of Boron and Exogenous Hormones on Fruit Yield

**Figure 6:**
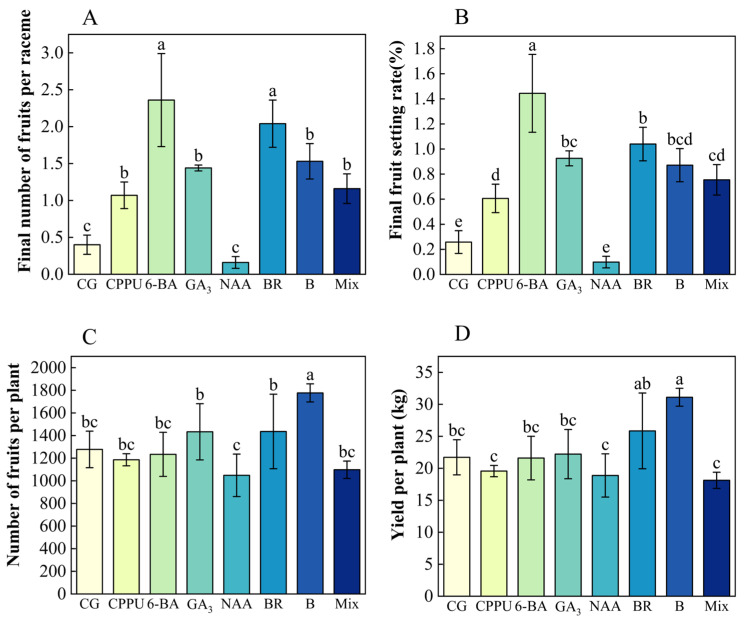
Effects of boron fertilizer and exogenous hormones on nut production. (**A**) Final fruit set (number per raceme); (**B**) final fruit setting rate (%); (**C**) total fruit number under different treatments; (**D**) yield per plant (kg). Data represent means ± SD (n = 3). Different letters (a, b, c, d, e) indicate significant differences among groups (*p* < 0.05, Duncan’s test).

To evaluate the effects of boron fertilizer and exogenous hormones on fruit yield, key parameters were measured, including whole-plant yield, fruit number per plant, final fruit count per raceme, and fruit setting rate at maturity. Among the treatments, 6-BA and BR proved most effective, increasing fruit numbers by 490% and setting rates by 476% compared to the control. Other treatments—CPPU, boron, GA_3_, and a mixed-solution—also significantly enhanced final fruit setting rate, whereas NAA consistently underperformed (Figure 6A,B). Notably, boron treatment exhibited the strongest impact on productivity, yielding the highest field output (31.1 kg/tree, a 43.11% increase over the control) and the greatest fruit number per tree (39.12% higher than the control) (Figure 6C,D).

## 3. Discussion

Previous studies have demonstrated that boron fertilizer application and exogenous hormone treatment significantly regulate fruit yield formation and quality improvement in economic crops such as pepper, tomato, and pomegranate [56,57]. In this study, boron fertilizer and exogenous hormones significantly affected fruit yield and quality. Among the treatments, 6-BA resulted in the highest fruit setting rate, while boron fertilizer maximized yield, fruit count, and economic value. BR led to the largest fruit size and highest weight. Boron notably boosted yield, consistent with prior studies on tomato [58] and olive [59]. BR also effectively regulated fruit growth [60]. Studies have shown that the application of cytokinins (e.g., CPPU and 6-BA) during early fruit development significantly enhances initial fruit set, with CPPU exhibiting the most pronounced effect. This finding aligns with previous research on persimmon and litchi, further confirming the positive role of cytokinins in promoting fruit set [61,62]. GA_3_ minimized early fruit abscission (Figure 3D), a result supported by an apple study [63], while boron enhanced fruit diameter, as seen in studies on bayberry and pomegranate [64,65]. NAA’s low retention rate may stem from the use of an excessive concentration (20 mg/L), which contrasts with its thinning effect [66] or benefits at lower doses [28]. This concentration-dependent effect underscores the need for precise hormone dosing in orchard management, particularly given macadamia’s sensitivity to synthetic auxins.

Boron fertilizer and exogenous hormones have been proven to play important regulatory roles in the development of flowers [67,68]. In this study, exogenous regulators altered pollen viability, raceme length and peduncle length, and flower number. Boron fertilizer treatment resulted in the highest pollen viability and longest racemes, while the mixed-solution produced the thickest racemes. Previous studies indicate that boron promotes pollen germination in cherries and lilies [69,70] and is vital for raceme development [71]. BR, a growth promoter in lotus [72], increased macadamia’s flower number per raceme the most. These floral modifications are agriculturally significant: longer racemes improve pollinator accessibility, while higher pollen viability ensures effective fertilization—both factors directly contributing to the high outcrossing rates required for premium macadamia yield [73]. During the new shoot period, boron and hormones affected leaf size, shape, and shoot length. GA_3_ produced the longest leaves, while 6-BA led to the widest leaves. CPPU and BR treatments resulted in the highest and lowest leaf length-to-width ratios, respectively. NAA produced the longest shoots, whereas BR yielded the shortest. NAA most effectively promoted shoot growth, consistent with its positive effects on other plants, such as sugarcane [74]. From an industry perspective, shoot vigor modulation through hormone applications could optimize orchard light interception and spatial arrangement.

Previous studies have shown that boron fertilizer and exogenous hormones have significant regulatory effects on soluble sugars, proteins, and endogenous hormones in plants. Specifically, NAA application reduces sucrose content in peach fruits, whereas gibberellin enhances sugar accumulation [75]. In olive trees, endogenous gibberellin levels rise with increasing boron concentrations after boron fertilization [76]. Additionally, soluble protein content is markedly elevated in mulberry trees treated with boron [77]. In this study, BR, the mixed-solution, and 6-BA significantly increased soluble sugar content in flowers at the initial flowering stage. However, only BR maintained higher levels at the peak flowering stage compared to the control. Similarly, GA_3_, CPPU, and 6-BA initially boosted soluble protein content, while 6-BA, BR, GA_3_, and NAA showed higher levels than the control at peak flowering. Previous studies suggest that BR enhances stress tolerance by increasing soluble sugars and proteins in drought- or pollution-affected plants [78,79]. Thus, hormone sprays may improve macadamia’s stress tolerance during early flowering, thereby increasing fruit set. This is particularly relevant for expanding cultivation in marginal areas (e.g., water-limited regions), where climate resilience directly impacts yield stability. Cytokinins are critical for fruit development [80]. In this study, boron fertilizer, 6-BA, and BR significantly raised cytokinin-like hormone levels during peak flowering, likely contributing to improved initial fruit set. GA_3_-treated plants exhibited higher GA_3_ content in flowers (Table 2) and lower fruit abscission rates, consistent with findings that GA_3_ prolongs fruit retention in citrus [81]. IAA levels rose under 6-BA, mixed-solution, and boron fertilizer treatments at the initial flowering stage, and remained elevated under mixed-solution, BR, 6-BA, and GA_3_ at the peak flowering stage, correlating with higher fruit set rates. Since auxin is vital for fruit development [82], its increase may enhance macadamia fruit set. JA content increased under 6-BA at the initial flowering stage and under CPPU/mixed-solution at the peak flowering stage, coinciding with higher initial fruit set rates. JA regulates plant growth, pollen development, and stress resistance [83,84], suggesting that these treatments improve fruit set via JA-mediated effects. New mechanistic insights reveal that JA orchestrates mitochondrial retrograde signaling through the *COI1*-*MYC2*-*ERF109* module, balancing growth and defense under stress [85]. This implies that JA elevation in our treatments may simultaneously bolster pest/disease resistance—a key consideration for reducing pesticide use in sustainable orchards. For example, JA-induced defense could mitigate damage from macadamia nut borer or husk spot disease.

The boron–hormone regulatory pattern identified in this study provides technical support for macadamia cultivation practices across diverse varieties, planting regions, and interannual variations. Consistent with our findings, Zeng et al. (2014) demonstrated in the Zhanjiang region (a province in China) that the application of CPPU effectively reduced early fruit drop in ‘Namya 2’ (Macadamia integrifolia) [39], further substantiating the applicability of this regulatory pattern. Trueman (2010) reported that applying BA to macadamia flowers or young fruit increased initial fruit set and delayed abscission but did not significantly improve final yield [86]. Although our 6-BA experiment aligned with Trueman’s results regarding initial fruit set, we observed a significant increase in final fruit set. This discrepancy may be attributed to genotype-specific or environment-specific factors, requiring further validation. Similarly, Silva et al. (2018) observed that boron spraying enhanced initial fruit set in Australian varieties ‘842’ and ‘A4’ [86], yet their treatment failed to increase final yield, potentially due to soil–climate interactions. These inconsistencies highlight that the universality of our findings may be constrained by genotype–environment interactions and interannual climate variability.

Based on our findings, we propose an integrated model in which the dynamic regulation of endogenous auxin (IAA) acts as the central hub, coordinating with other hormonal signals to collectively modulate macadamia fruit set. This model highlights that exogenous hormones (e.g., 6-BA) and boron fertilizer enhance early fruit setting potential by elevating IAA levels in floral organs, thereby directly promoting ovary development and nutrient allocation (e.g., soluble sugar and protein accumulation). Cytokinin (6-BA) synergizes with auxin to activate meristematic activity, increasing initial fruit set, while gibberellin reduces fruit abscission by delaying abscission layer formation. The rise in JA may balance fruit set with stress resistance under challenging environments (e.g., drought) by regulating both defense responses and reproductive growth. Notably, the effects of these hormones vary across flowering stages, necessitating precise hormone application tailored to specific developmental phases. This model provides a theoretical foundation for stabilizing macadamia yields: targeted modulation of endogenous hormone gradients and downstream metabolic pathways can optimize both fruit set and environmental adaptability while reducing reliance on chemical pesticides. Future studies should further elucidate the spatiotemporal dynamics of hormonal signaling at the cell type level.

## 4. Materials and Methods

### 4.1. Experimental Materials

This study was conducted at the Lujiangba research base of the Institute of Tropical and Subtropical Economic Crops, Yunnan Academy of Agricultural Sciences, located in Longyang District, Baoshan City, Yunnan Province (24°58′22″ N, 98°52′51″ E; elevation 645.5 m). Situated in the southwestern region of Yunnan Province, China, this area is characterized by low-altitude plateaus within the longitudinal valleys of the Hengduan Mountain Range. The Nujiangba basin features hills interspersed with dam areas, abundant sunlight, a year-round frost-free climate, and an average annual temperature of 25 °C. As a representative subtropical dry–hot valley ecosystem, it provides an optimal environment for cultivating various economic crops. The presence of abundant macadamia resources and extensive commercial orchards offered ideal conditions for this research.

The experiment utilized A4 variety macadamia trees with uniform growth characteristics and no visible pest or disease damage. All experimental trees were 11 years old, averaging 4.83 m in height, 11.58 cm in diameter at breast height (DBH), 12.86 cm in basal diameter, 4.48 m in canopy spread, and 25.38 mg/kg foliar boron concentration. Both treatment and control groups showed comparable growth parameters (Table S1), with no observable crown inclination in any specimens. Experimental treatments comprised the water control (CG), cytokinins (CPPU, and 6-BA, separately), gibberellin (GA_3_), auxin (NAA), boron fertilizer (B), brassinosteroids (BR), and a combination treatment containing all aforementioned compounds (MIX).

### 4.2. Spraying of Hormones and Boron Fertilizer Agents

A total of 24 macadamia trees (A4 variety) with uniform growth were selected for the experiment and divided into experimental and control groups. The experimental group received treatments with specific plant growth regulators and boron fertilizer, while the control group was sprayed with water only. The treatments for the experimental group included the following: 80.7 μM CPPU; 8.88 μM 6-BA; 57.8 μM GA_3_; 107 μM NAA; 0.416 μM BR; 500 μM B; and a mixed-solution containing all components at specified concentrations (80.7 μM CPPU + 8.88 μM 6-BA + 57.8 μM GA_3_ + 107 μM NAA + 0.416 μM BR + 500 μM B). Due to the large tree size, each treatment was applied to three trees as replicates. Applications were administered on the whole canopy during three growth stages, flower bud stage (February), full flowering (March), and young fruit formation (post-flowering, April), totaling three sprays per treatment. Spraying was conducted on sunny mornings, and after application, water droplets were observed on leaf surfaces.

All chemicals were purchased from commercial suppliers: boron fertilizer from U.S. Borax (Harbin, China); CPPU from Sichuan Guoguang Agrochemical Co., Ltd. (Chengdu, China); GA_3_ from Zhejiang Qianjiang Biochemical Co., Ltd. (Haining, China); NAA from Jiangxi New Reyphon Biochemical Co., Ltd. (Ji’an, China); brassinolide from Chengdu New Sun Crop Science Co., Ltd. (Chengdu, China); and 6-BA from Zhejiang Dapeng Pharmaceutical Co., Ltd. (Taizhou, China).

### 4.3. Flower Bud and Leaf Collection and Leaf Morphology Determination

Following the initial hormone treatment, ten racemes were randomly collected from each tree using a long-handled pruner. Leaf samples were collected at three key phenological stages: flowering initiation, peak flowering, and post-flowering. From each tree, mature leaves were harvested from the second and third youngest nodes of newly grown branches. Specifically, five branches were selected per tree, with two leaves sampled from each branch (resulting in ten leaves per tree). All samples were immediately wrapped in aluminum foil and stored at −80 °C for subsequent biochemical analysis. Additionally, ten leaves were collected following the identical protocol and their dimensions (length and width) were measured using a calibrated ruler.

### 4.4. Pollen Viability Determination

Pollen viability was determined using the agar culture method [17]. At the looping stage, flowers exhibiting arched styles and freshly cracked anthers were collected. Two flower spikes were obtained from each tree. After removing the anthers to release the pollen, the pollen was immediately cultured. A culture medium was prepared, consisting of 0.5% agar and 10% sucrose, and adjusted to a pH of 5.8–6.0. The pollen was transferred onto glass slides coated with agar and placed in a Petri dish. To maintain humidity, the dish lid was lined with water-absorbing filter paper. The samples were then incubated in a light incubator at 25 °C for 10 h. After incubation, the pollen was examined under a microscope at 10 × 16 magnification. For each treatment, three slides were prepared, and five fields of view were observed per slide to determine the pollen germination rate.

### 4.5. Flower and Leaf Collection at Full Bloom

During the full bloom period, ten racemes and leaves were collected from each tree after the second hormone application. The sampled flowers and leaves were immediately wrapped in aluminum foil and stored at −80°C for subsequent biochemical analysis.

### 4.6. Fruit Investigation and Collection

During the investigation, 30 racemes were randomly marked on each tree. The number of flowers per raceme was recorded at full bloom. Fruit abscission dynamics were monitored by counting the number of abscised fruits on the marked racemes at 1, 2, 3, 4, and 20 weeks after initial fruit set. Data collection was conducted in the morning on sunny days: the number of retained fruits per raceme was recorded and averaged across the racemes, with this average representing the mean fruit set per raceme for that tree in the given week. The mean fruit set per raceme recorded in the first week after initial fruit set was designated as the initial fruit set per raceme, with the difference in fruit set per raceme between consecutive weeks representing the weekly fruit abscission per raceme, which was divided by the initial fruit set per raceme to calculate the weekly fruit abscission rate. The periods of 1–3 weeks, 4–20 weeks, and 1–20 weeks after initial fruit set were classified as the first batch, second batch, and total fruit abscission, respectively, with the number and rate of fruit abscission being calculated for each period.

At week 20, the final fruit set per raceme was determined, and sampling was conducted by randomly selecting ten racemes from different positions on the tree and harvesting all fruits from these racemes to measure fruit quality traits including fresh nut-in-shell mass, fresh nut-in-husk mass with pericarp, fruit transverse diameter, and fruit longitudinal diameter. The sampled fruits were dried in an oven at 50 °C until constant mass was achieved, after which the dry mass of nuts and kernel mass were measured to calculate the aspect ratio, nut-in-shell recovery, and kernel recovery. Yield measurement was performed in the afternoon on a sunny day at week 22 by harvesting all green-husked fruits from each tree, immediately placing them in individual bags for weighing, and recording the total mass as the yield per tree. The calculation formulas are as follows:

The fruit abscission rate = (Initial fruit set number per raceme − Final fruit set number per raceme)/Initial fruit set per raceme × 100%(1)

The fruit set rate = The average number of fruits per raceme/The average number of flowers per raceme × 100%(2)

Aspect ratio = Longitudinal diameter/transverse diameter(3)

Nut-in-shell recovery = Fresh nut-in-shell mass/Fresh nut-in-husk mass × 100%(4)

Kernel recovery = Kernel mass/Oven-dry nut-in-shell mass × 100%(5)

### 4.7. New Shoot Growth Monitoring

New shoot growth assessments were conducted in mid-May. The survey included measurements of the following parameters: leaf dimensions (including newly sprouted leaf length, width, and the length-to-width ratio) and shoot morphology (such as total new shoot length, node count, and internode length, measured as the distance between the first two nodes). The new shoot survey was conducted on a sunny morning. For each tree, 3 new shoots in different directions were randomly selected for investigation. For each new shoot, the leaf length and width of the 2 largest leaves were measured, the total new shoot length and the internode length of the first node were measured, and the number of nodes of the new shoot and the leaf length-to-width ratio were counted.

### 4.8. Determination of Endogenous Hormones and Physiological Indicators

Endogenous hormone levels were quantified using high-performance liquid chromatography (HPLC) coupled with a triple quadrupole liquid chromatography–mass spectrometer (LC-MS/MS). Whole floral organs (excluding pedicels) were collected as experimental materials for hormone quantification. Briefly, approximately 200 mg of fresh tissue samples was homogenized in liquid nitrogen and extracted with 1 mL of an aqueous acetonitrile solution (H_2_O/ACN, 90:10, *v*/*v*) that was spiked with internal standards prior to extraction. After centrifugation at 4 °C for 20 min, the supernatant was collected and the residue was re-extracted following the same procedure. The combined extract was then derivatized with 10 µL of triethylamine and 10 µL of 3-propyl trimethyl ammonium bromide, followed by vortexing and incubation at 90 °C for 1 h. The reaction mixture was dried under nitrogen gas and reconstituted in 100 µL of H_2_O/ACN for subsequent LC-MS analysis.

Soluble sugar content was measured using the anthrone colorimetric method [87], and soluble protein content was determined using the Coomassie brilliant blue method [88]. The analyses were performed on whole floral organs (excluding pedicels) and leaves.

### 4.9. Statistical Methods

Each treatment in this study contains three replicates, where each replicate records the mean value of 15 samples and calculates the mean value and standard deviation of the three replicates. One way ANOVA and multiple comparisons are performed between different treatments. All data were analyzed by R4.3.2 program, ANOVA and Duncan test were used for multi-group comparison between different treatments, the statistical significance was set as *p* < 0.05 (bilateral test), and the quantitative data was expressed as [mean ± standard deviation].

## 5. Conclusions

This study investigated the effects of various exogenous hormones and boron fertilizers applied via leaf spraying on different growth stages and their impact on yield. The research analyzed physiological indicators and hormonal responses to elucidate the regulatory mechanisms, leading to the proposal of an optimal spraying regimen. Key findings include the following: At the flowering stage, spraying 0.02% boron fertilizer enhanced pollen viability, raceme length, and flower quantity. During peak flowering, applying 2 mg/L of 6-BA improved fruit set rate. From the withering to early fruit setting stage, a combination of 0.02% boron fertilizer and 0.2 mL/L of BR increased yield, reduced fruit abscission, and promoted larger fruit size. Physiological analysis revealed that elevated soluble sugars and proteins in flowers contributed to higher initial fruit set. Additionally, boron fertilizer treatment likely boosted yield by increasing endogenous IAA levels during early flowering. Furthermore, higher endogenous cytokinin levels in flowers improved initial fruit set, while increased GA_3_ content helped lessen abscission. Collectively, these results provide a science-based approach to optimizing nut yield through targeted growth-stage interventions. These findings are specific to the ‘A4’ cultivar under the experimental conditions of a single site and year. The generalizability of these findings to other cultivars, growing years, or geographical regions may be limited and requires further validation in future studies.

## Figures and Tables

**Table 1:** New shoot traits under different treatments. Data represent means ± SD (n = 3). Different letters (a, b, c) indicate significant differences among groups (*p* < 0.05, Duncan’s test).

Treatment	Leaf Length (cm)	Leaf Width (cm)	Length-to-Width Ratio	Number of Nodes (pcs)	Shoot Length (cm)	First Internode Length (cm)
CG	7.23 ± 1.98 c	1.89 ± 0.53 c	3.83 ± 0.22 abc	2.33 ± 0.33 a	6.76 ± 0.53 ab	2.82 ± 0.30 a
CPPU	7.92 ± 1.23 abc	1.99 ± 0.48 bc	4.05 ± 0.49 a	2.00 ± 0.00 a	6.69 ± 0.97 ab	2.79 ± 0.39 a
6-BA	9.50 ± 0.48 ab	2.68 ± 0.13 a	3.54 ± 0.22 abc	2.00 ± 0.00 a	6.09 ± 0.80 bc	2.48 ± 0.28 a
GA_3_	10.11 ± 1.03 a	2.53 ± 0.22 ab	4.01 ± 0.41 ab	2.00 ± 0.00 a	6.81 ± 0.70 ab	2.78 ± 0.21 a
NAA	8.46 ± 0.83 abc	2.13 ± 0.21 abc	3.98 ± 0.12 abc	2.89 ± 0.69 a	7.39 ± 0.68 a	2.71 ± 0.56 a
BR	8.42 ± 0.15 abc	2.52 ± 0.12 ab	3.35 ± 0.21 c	2.00 ± 0.00 a	5.29 ± 0.39 c	1.98 ± 0.28 a
B	9.50 ± 1.63 ab	2.45 ± 0.23 abc	3.86 ± 0.32 abc	2.33 ± 0.33 a	5.32 ± 0.76 c	2.08 ± 0.61 a
Mix	7.72 ± 0.74 bc	2.29 ± 0.28 abc	3.39 ± 0.45 bc	2.44 ± 1.07 a	6.51 ± 0.22 abc	2.30 ± 1.01 a

**Table 2:** Endogenous hormones (unit: ng/g). Data represent means ± SD (n = 3). Different letters (a, b, c, d, e) indicate significant differences among groups (*p* < 0.05, Duncan’s test).

Stage	Hormone	CG	CPPU	6-BA	GA_3_	NAA	BR	B	Mix
The initial flowering stage	ZEA	0.026 ± 0.003 bc	0.01 ± 0.003 d	0.038 ± 0.003 a	0.04 ± 0.003 a	0.024 ± 0.003 c	0.03 ± 0.004 b	0.023 ± 0.003 c	0.003 ± 0.000 e
	GA_1_	2.59 ± 0.24 a	0.91 ± 0.05 c	0.57 ± 0.03 d	1.34 ± 0.14 b	1.43 ± 0.17 b	0.58 ± 0.04 d	0.79 ± 0.09 c	0.91 ± 0.01 c
	GA_3_	0.008 ± 0.002 c	0.007 ± 0.002 c	0.003 ± 0.000 c	0.338 ± 0.011 b	0.007 ± 0.001 c	0.005 ± 0.002 c	0.005 ± 0.002 c	0.478 ± 0.045 a
	IAA	6.87 ± 0.12 c	6.03 ± 1.01 c	19.33 ± 1.26 a	5.55 ± 0.71 c	5.06 ± 1.44 c	5.41 ± 0.61 c	9.47 ± 1.13 b	5.19 ± 0.46 c
	ICA	0.63 ± 0.10 cd	0.45 ± 0.04 de	0.61 ± 0.15 d	0.32 ± 0.00 e	0.61 ± 0.02 d	0.82 ± 0.14 c	1.18 ± 0.11 b	1.54 ± 0.18 a
	ABA	16.87 ± 2.21 e	46.35 ± 5.55 b	31.48 ± 0.28 c	21.13 ± 4.01 e	57.07 ± 1.81 a	31.96 ± 0.33 c	26.57 ± 0.13 d	47.97 ± 0.62 b
	JA	159.4 ± 20.44 bcd	144.89 ± 3.47 cd	246.14 ± 39.63 a	113.58 ± 23.90 d	184.08 ± 12.94 bc	126.94 ± 30.35 d	201.89 ± 36.08 ab	182.47 ± 19.68 bc
	SA	10.79 ± 0.13 cd	15.49 ± 2.2 ab	10.85 ± 0.18 cd	13.07 ± 2.66 bc	8.89 ± 0.52 de	11.76 ± 0.43 cd	7.85 ± 1.66 e	17.84 ± 2.01 a
The peak flowering stage	ZEA	0.047 ± 0.002 c	0.028 ± 0.003 d	0.062 ± 0.002 a	0.049 ± 0.003 bc	0.028 ± 0.006 d	0.054 ± 0.003 b	0.044 ± 0.002 c	0.009 ± 0.002 e
	GA_1_	0.09 ± 0.01 b	0.09 ± 0.00 b	0.04 ± 0.00 d	0.3 ± 0.00 a	0.04 ± 0.01 d	0.03 ± 0.01 e	0.04 ± 0.01 de	0.06 ± 0.01 c
	GA_3_	0.009 ± 0.004 c	0.004 ± 0.001 c	0.009 ± 0.003 c	7.883 ± 0.792 a	0.037 ± 0.012 c	0.019 ± 0.006 c	0.051 ± 0.002 c	7.102 ± 0.696 b
	IAA	2.83 ± 0.87 c	2.09 ± 0.57 c	5.76 ± 0.93 a	4.32 ± 1.08 b	1.92 ± 0.22 c	5.98 ± 1.00 a	1.45 ± 0.17 c	6.93 ± 0.54 a
	ICA	0.54 ± 0.00 bc	0.51 ± 0.07 bc	0.54 ± 0.07 bc	0.45 ± 0.01 c	0.4 ± 0.07 c	0.63 ± 0.10 b	0.44 ± 0.10 c	0.91 ± 0.10 a
	ABA	41.51 ± 1.17 bc	43.6 ± 0.76 b	45.67 ± 7.03 b	34.19 ± 2.32 c	48.62 ± 4.9 ab	55.18 ± 4.07 a	35.51 ± 4.78 c	46.76 ± 5.09 b
	JA	67.43 ± 2.22 cde	105.22 ± 15.99 a	63.88 ± 2.94 cde	71.66 ± 0.83 bcd	54.54 ± 5.11 e	76.75 ± 8.31 bc	60.23 ± 7.35 de	84.59 ± 1.26 b

## Data Availability

No new data were created or analyzed in this study.

## Supplementary Materials

The following supporting information can be downloaded at: https://www.mdpi.com/article/10.3390/plants14162461/s1, Table S1: Morphological characteristics of experimental trees. Table S2: Leaf morphology. Figure S1: Fruit set and abscission dynamics per raceme during first four weeks post-flowering. (A) Fruit quantity in second week; (B) fruit quantity in third week; (C) fruit quantity in fourth week; (D) fruit abscission in first week; (E) fruit abscission in second week; (F) fruit abscission in third week. Figure S2: Fruit diameter growth (mm) during first four weeks after different treatments. (A) Diameter at initial fruit set (first week); (B) diameter at second week; (C) diameter at third week; (D) diameter at fourth week.

## Abbreviations

The following abbreviations are used in this manuscript:

CG	Control group (water control)
B	Boron fertilizer
BR	Brassinosteroids
CPPU	N-(2-Chloro-4-pyridyl)-N’-phenylurea
6-BA	6-benzylaminopurine
GA3	Gibberellic acid
NAA	α-Naphthaleneacetic acid
HPLC	High-performance liquid chromatography
LC-MS/MS	A triple quadrupole liquid chromatography–mass spectrometer
