# Supplementary material for: Boron Supplementation and Phytohormone Application: Effects on Development, Fruit Set, and Yield in Macadamia Cultivar ‘A4’ (Macadamia integrifolia, M. tetraphylla)
Source: Plants (Basel). 2025 Aug 8;14(16):2461. doi: 10.3390/plants14162461 (PMC12389283; doi:10.3390/plants14162461)
Supplement: Supplementary file 1 [file plants-14-02461-s001.zip › plants-3764101-supplementary.pdf]

plants

MDPI

Table S1. Morphological characteristics of experimental trees. Data represent means ± SD (n = 3). Different letters (a, b) indicate significant differences among groups ($p < 0.05$, Duncan's test).

Treatment	Tree height (m)	Diameter at breast height (cm)	Basal diameter (cm)	East-west canopy spread (m)	North-south canopy spread (m)	Branch height (m)
CK	4.73 ± 0.49 a	11.53 ± 0.68 a	12.73 ± 1.15 a	4.34 ± 0.17 ab	4.31 ± 0.40 ab	69.00 ± 18.19 a
CPPU	4.87 ± 0.59 a	11.37 ± 0.64 a	12.90 ± 0.78 a	4.47 ± 0.52 ab	4.46 ± 0.58 ab	71.33 ± 19.04 a
6-BA	4.80 ± 0.20 a	11.43 ± 0.40 a	12.43 ± 0.57 a	4.31 ± 0.07 ab	4.52 ± 0.17 ab	48.00 ± 19.47 a
GA_3	5.30 ± 0.72 a	11.73 ± 0.59 a	12.53 ± 0.15 a	4.61 ± 0.58 ab	4.61 ± 0.58 ab	54.67 ± 13.43 a
NAA	4.80 ± 0.30 a	11.27 ± 0.31 a	12.50 ± 0.20 a	4.57 ± 0.04 ab	4.51 ± 0.15 ab	53.33 ± 8.96 a
BR	4.57 ± 0.15 a	11.70 ± 0.46 a	13.17 ± 0.57 a	4.57 ± 0.20 ab	4.55 ± 0.27 ab	44.67 ± 14.01 a
B	5.13 ± 0.15 a	11.77 ± 0.32 a	12.83 ± 0.59 a	4.92 ± 0.18 a	4.86 ± 0.13 a	52.33 ± 33.98 a
Mix	4.67 ± 0.72 a	11.80 ± 0.70 a	13.30 ± 0.69 a	4.06 ± 0.32 b	4.14 ± 0.29 b	45.00 ± 15.00 a

Table S2. Leaf Morphology. Data represent means ± SD (n = 3). Different letters (a, b) indicate significant differences among groups ($p < 0.05$, Duncan's test).

Treatment	Leaf Length (cm)	Leaf Width (cm)	Leaf Length to Width Ratio (cm)
CK	12.40 ± 0.38 a	3.19 ± 0.12 a	3.88 ± 0.18 a
CPPU	12.42 ± 0.29 a	3.45 ± 0.26 a	3.64 ± 0.22 a
6-BA	12.39 ± 1.05 a	3.25 ± 0.16 a	3.83 ± 0.10 a
GA_3	12.60 ± 0.86 a	3.46 ± 0.35 a	3.66 ± 0.16 a
NAA	12.44 ± 1.00 a	3.37 ± 0.21 a	3.73 ± 0.56 a
BR	12.55 ± 0.61 a	3.58 ± 0.10 a	3.53 ± 0.13 a
B	12.87 ± 1.70 a	3.51 ± 0.35 a	3.66 ± 0.11 a
Mix	12.33 ± 1.13 a	3.26 ± 0.38 a	3.85 ± 0.20 a

Note: No significant differences in macadamia leaf morphology were observed across treatment groups.

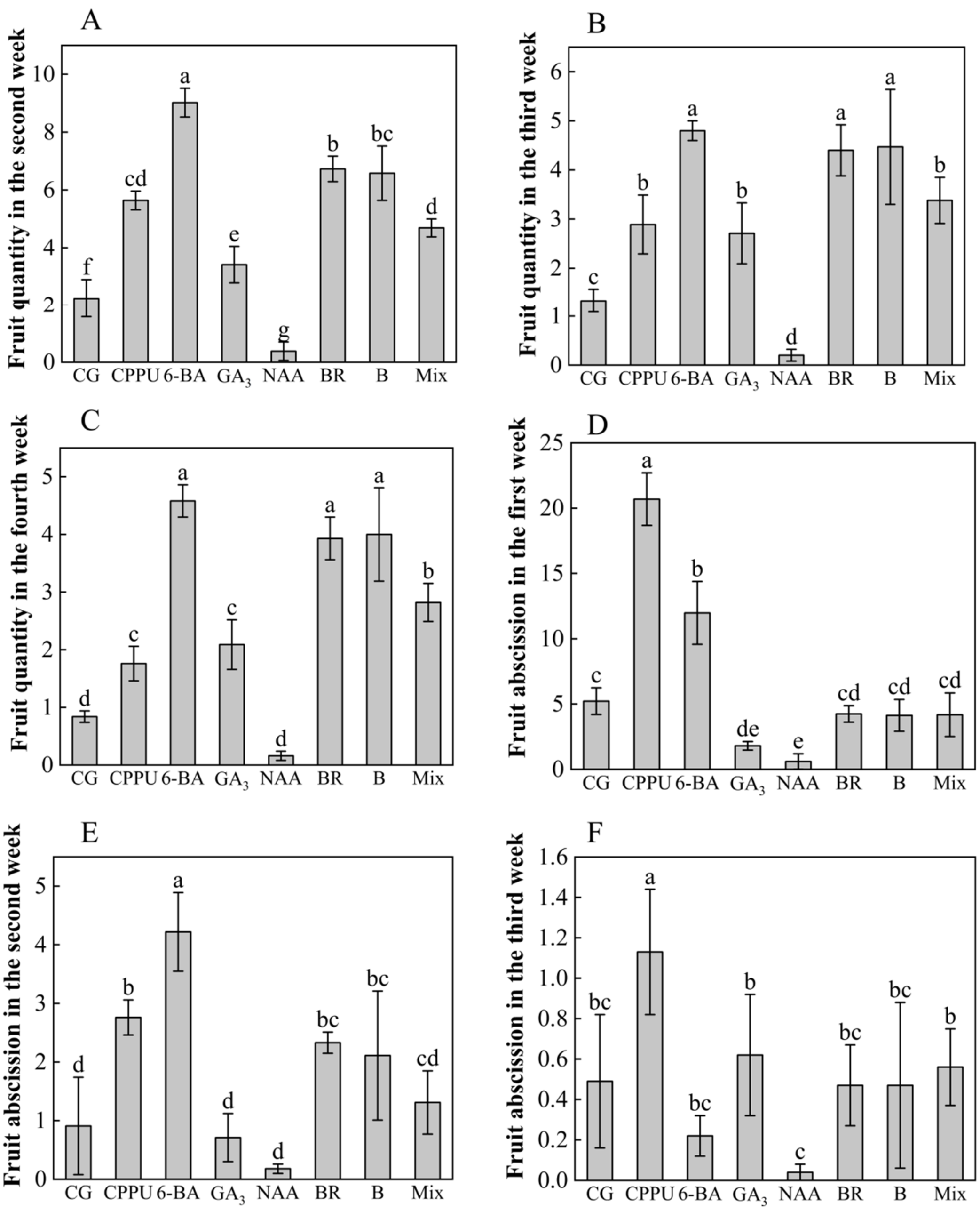

Figure S1: Fruit set and abscission dynamics per raceme during the first four weeks post-flowering. (A) Fruit quantity in the second week; (B) fruit quantity in the third week; (C) fruit quantity in the fourth week; (D) fruit abscission in the first week; (E) fruit abscission in the second week; (F) fruit abscission in the third week. Data represent means ± SD (n = 3). Different letters (a, b, c, d, e, f, g) indicate significant differences among groups ($p < 0.05$, Duncan's test).

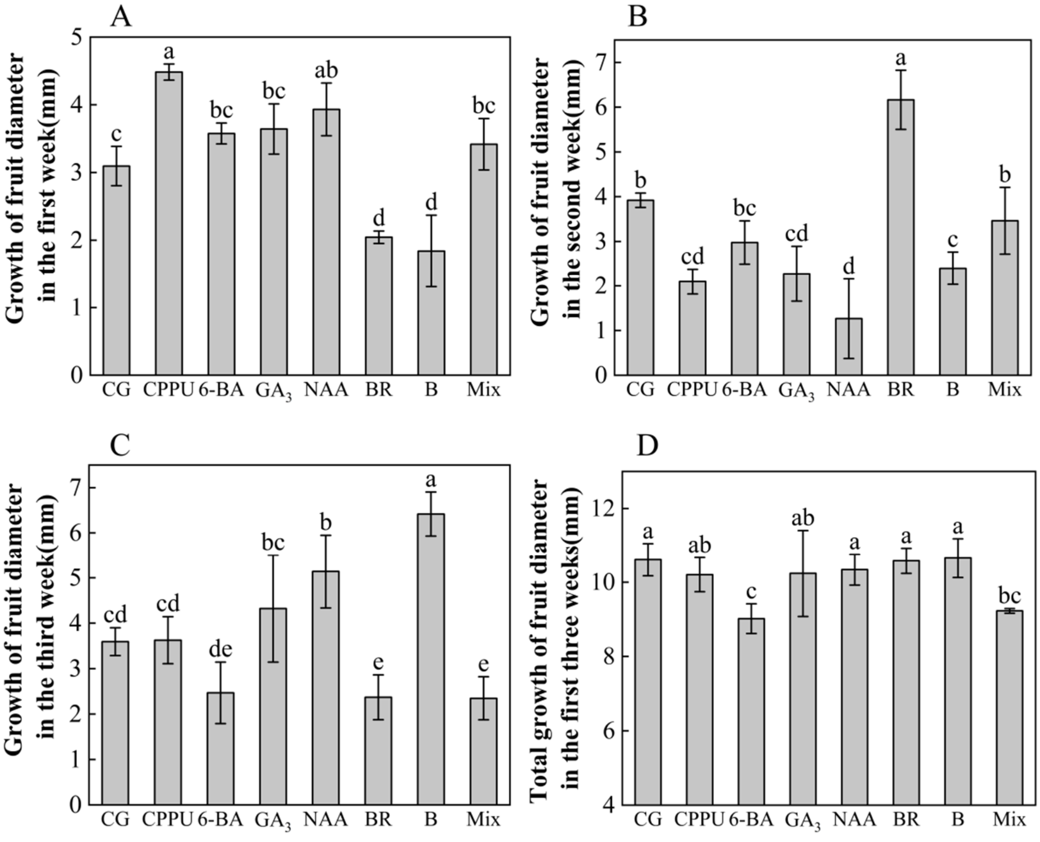

Figure S2. Fruit diameter growth (mm) during the first four weeks after different treatments. (A) Diameter at initial fruit set (first week); (B) diameter at second week; (C) diameter at third week; (D) diameter at fourth week. Data represent means ± SD (n = 3). Different letters (a, b, c, d, e) indicate significant differences among groups ($p < 0.05$, Duncan's test).
